# Dynamic proteome allocation regulates the profile of interaction of auxotrophic bacterial consortia

**DOI:** 10.1098/rsos.212008

**Published:** 2022-05-04

**Authors:** D. Reyes-González, H. De Luna-Valenciano, J. Utrilla, M. Sieber, R. Peña-Miller, A. Fuentes-Hernández

**Affiliations:** ^1^ Synthetic Biology Program, Center for Genomic Sciences, Universidad Autónoma de México, 62220 Cuernavaca, Mexico‌‌; ^2^ Systems Biology Program, Center for Genomic Sciences, Universidad Nacional Autónoma de México, 62210 Cuernavaca, Mexico; ^3^ Max Planck Institute for Evolutionary Biology, 24306 Plön, Germany

**Keywords:** microbial communities, mathematical modelling, experimental microbiology

## Abstract

Microbial ecosystems are composed of multiple species in constant metabolic exchange. A pervasive interaction in microbial communities is metabolic cross-feeding and occurs when the metabolic burden of producing costly metabolites is distributed between community members, in some cases for the benefit of all interacting partners. In particular, amino acid auxotrophies generate obligate metabolic inter-dependencies in mixed populations and have been shown to produce a dynamic profile of interaction that depends upon nutrient availability. However, identifying the key components that determine the pair-wise interaction profile remains a challenging problem, partly because metabolic exchange has consequences on multiple levels, from allocating proteomic resources at a cellular level to modulating the structure, function and stability of microbial communities. To evaluate how ppGpp-mediated resource allocation drives the population-level profile of interaction, here we postulate a multi-scale mathematical model that incorporates dynamics of proteome partition into a population dynamics model. We compare our computational results with experimental data obtained from co-cultures of auxotrophic *Escherichia coli* K12 strains under a range of amino acid concentrations and population structures. We conclude by arguing that the stringent response promotes cooperation by inhibiting the growth of fast-growing strains and promoting the synthesis of metabolites essential for other community members.

## 1. Introduction

Bacterial cells rarely live in isolation, but coexist in communities composed of a genetically diverse assembly of different individuals, with interactions mediated by the exchange of small molecules. Genetically diverse populations tend to exhibit increased resilience to invasion [1] and to environmental perturbations [2–4], as well as to enable the implementation of division of labour strategies [5–8]. In particular, cross-feeding interactions can increase the diversity of the community and lead to metabolic cooperation [9,10] by changing the population’s dynamics and composition [1,11]. If the consumption of a metabolite benefits one of the interacting partners and is detrimental for the other, it is referred to as parasitism [12], while if it also benefits the metabolite-producing population, then the community is said to be *mutualistic* [13,14].

Crucially, metabolic exchange mediated by the environment is not always associated with a benefit for the community [15,16]. Moreover, metabolic interactions are not static but condition specific [17–20], resulting from metabolic shifts [21] and trade-offs between different microbial growth traits [22]. Previous studies have shown that in high-nutrient environments, diversity is reduced and interactions tend to be antagonistic [2,16,23], thus increasing susceptibility to invasion by defectors [24,25]. By contrast, nutrient obligate cross-feeding interactions have been shown to become more cooperative in low-nutrient environments, both in natural communities [26] and in synthetic systems [2,18,27–29].

But a key cellular process occurs under amino acid starvation. In environments where nutrients are scarce, cells regulate their global metabolism to shut down growth and increase the synthesis of limiting metabolites [30,31]. This dynamic partition of the proteome is mediated by two small nucleotides collectively referred to as ppGpp. This alarmone is known to modify the expression of hundreds of genes [32,33], including those responsible for the synthesis of the translation machinery [34]. Indeed, altering the normal levels of ppGpp was shown to produce several growth impairments and lethal phenotypes [35]. On the other hand, ppGpp regulation enables a dynamic proteome allocation that can be used to tune the concentration of ribosomes to maximize growth [36] and increase adaptation to fluctuating environments [37]. Furthermore, the stringent response is also known to increase survival to nutrient starvation [38,39], by allocating a large fraction of the proteome towards metabolite production at the expense of ribosomal synthesis [38,40–42].

The objective of this paper is to evaluate how dynamic proteome allocation in response to nutrient limitation impacts the population dynamics of a syntropic community. The paper is structured as follows. First, we will postulate a dynamic proteome partition model that provides estimates on growth and amino acid production rates, which are then used by a population dynamics model to evaluate how different environmental conditions result in different interaction profiles at a population level. We validate our theoretical predictions with an experimental model system consisting of different mutants of *Escherichia coli* K12 growing under a range of amino acid concentrations. This library of strains contains deletions in genes encoding for the production of essential amino acids, thus producing a syntropic interaction when grown in co-culture. Finally, we use the multi-scale model to evaluate computationally how ppGpp produces a dynamic proteome allocation that can drive the diversity and productivity of a syntropic community.

## 2. Modelling bacterial growth under resource limitation

The development of genomic and bioinformatic tools has enabled researchers to comprehensively quantify the interaction between genes, proteins, reactions and metabolites, thus providing information that can be used to postulate and analyse genome-scale models [43]. These models aim to predict bacterial phenotypes by assuming an internal quasi-steady-state equilibrium and optimizing fluxes from the stoichiometry of every known metabolic reaction occurring within the cell. Multiple extensions have been proposed to evaluate how fluxes are exchanged between individuals, and from an individual to multiple interacting species [21,44–49]. By incorporating diffusion of cells and molecules in space and time, computational models based on dynamic flux balance analysis can be extended to include multiple bacterial types interacting through the environment [46,50].

The aim of the mathematical model postulated in this paper is to predict the population dynamics that emerge from the metabolic exchange between individuals in the community. This problem spans multiple levels of complexity: from metabolic regulation occurring at a cellular level in response to environmental perturbations (e.g. changes in biotic and abiotic conditions) to ecological constraints resulting from the interaction between cells and their local environment (e.g. uptake or secretion of nutrients). A wide range of mathematical modelling approaches have been used to study metabolic interactions in complex microbial communities, for instance by postulating mechanistic population dynamic models that can be used to evaluate the eco-evolutionary dynamics, although they are often limited by modelling and parametric uncertainties [51]. Other studies have posed coarse-grained models that associate growth rate with how bacterial cells allocate resources towards protein synthesis and metabolic functions [41,52–55].

**Figure 1.**
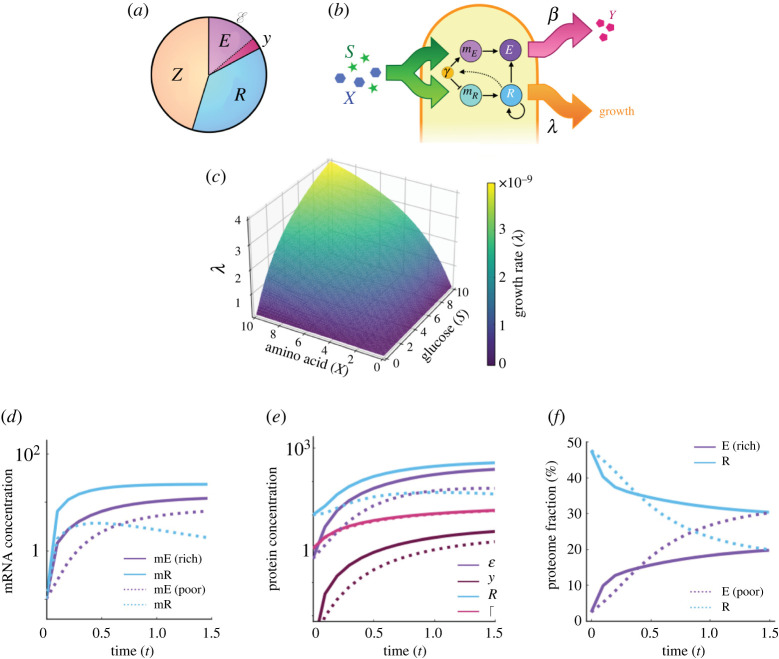
Proteome dynamics model. (*a*) The overall proteome comprises three main sectors: housekeeping proteins denoted as Z, ribosomal proteins as R and metabolism and nutrient uptake as E. The latter sector is further divided into E and *y* to consider the production of amino acid Y at a rate *β*. (*b*) Dynamics of proteome sectors are driven by the availability of glucose (*S*) and an essential amino acid (*X*). Growth rate is proportional to sector R, which in turn activates ppGpp during the stringent response, thus inhibiting transcription ribosomal mRNAs and promoting synthesis of metabolites from sector E. (*c*) Bacterial growth rate (*λ*) obtained numerically for a range of amino acid and glucose concentrations. (*d*) mRNA concentrations in time of different proteome sectors (purple for *m*_*E*_ and blue for *m*_*R*_) under different nutrient conditions: low-resource (*S* = 0.5, dotted lines) and rich substrate (*S* = 10, solid lines). (*e*) Protein concentrations for each sector (E in purple, *y* in dark red and R in blue). In environments with low concentrations of amino acids (indicated as poor, dotted lines), ppGpp (Γ, in pink) inhibits the production of ribosomal proteins and promotes the synthesis of metabolites. (*f*) Fraction of proteome occupied for different sectors (E in purple and R in blue). In high-nutrient environments (solid lines), a large proteome fraction is allocated for sector R, while in environments with low concentrations of amino acids (indicated as poor, dotted lines) the fraction allocated for sector E increases, thus inhibiting growth and promoting the production of the exchanged metabolite.

A common assumption of constraint-based models is that the proteome can be divided into three different functional sectors [40]: the ribosomal sector (hereafter denoted with R), which includes not only ribosomes, but also other associated molecules (initiation factors, elongation factors, tRNA synthases); the metabolic sector (represented with E), which encloses catabolic and anabolic proteins, constitutively expressed proteins, and particularly, the necessary enzymes to grow in specific media. As illustrated in figure 1*a*, we further divide sector E into two sub-sectors: *y* for the energetic cost of amino acid production, and E for the remaining catabolic and anabolic reactions occurring inside the cell. A third sector (denoted Z) encompasses all the genes not associated with growth rate, including housekeeping genes. Therefore, we consider that Z+(E+y)+R=1.

A recent study incorporated dynamic metabolic adaptation into a consumer-resource framework [56] to show that the structure and composition of competitive communities are highly influenced by the metabolism of member species [21,42]. Here, we use a similar approach to evaluate the role of dynamic proteome allocation in the resulting interaction profile exhibited by a co-culture of auxotrophic strains. First, as other studies [21,40,41], we assume that sector Z remains constant through time and we focus on evaluating the role of nutrient availability in the temporal dynamics of sectors E and R. Figure 1*b* shows a schematic diagram of our proteome partition model, illustrating that the size of each sector is regulated by the input of substrate (e.g. glucose, denoted by *S*) and an essential metabolite (e.g. an amino acid, with concentration denoted by *X*). We model the input of glucose and amino acid into the cell as a saturating function, which depends on both the concentration of *S* and *X*, which, for simplicity, we assume are consumed at the same rate:

$$u(S,X)=\frac{\mu (S\cdot X)}{k+(S\cdot X)},$$

where *μ* and *k* are parameters denoting the maximum uptake rates and half-saturation constants, respectively.

To overcome the negative effects associated with carbon and amino acid starvation, bacterial cells encode a well-conserved stress signalling pathway known as the stringent response [32,57]. The main effector of this response is the alarmone ppGpp, with a time-dependent concentration we denote by Γ(t). Crucially, synthesis and degradation of ppGpp depend on nutrient availability; if too many resources are being used to produce proteins for growth, *RelA* detects empty tRNAs and enhances the production of ppGpp, which in turn reshapes the proteome by enhancing the synthesis of metabolites from the E sector [57].

In our proteome partition model, we consider that ppGpp is produced at a rate *θ* = *θ*_*b*_ + *F*(*θ*_*i*_, *R*), where *θ*_*b*_ represents the basal transcription rate, and *F*(*θ*_*i*_; *R*) an inducible rate that increases monotonically with respect to *R*. So, if $\delta_{\Gamma }$ denotes a constant degradation rate of the alarmone, then the dynamics of ppGpp regulation can be modelled as

2.1 $$\frac{d\Gamma }{dt}=\theta_{b}+\frac{\theta_{i}\;R}{1+R}-\delta_{\Gamma }\Gamma .$$

To model the effect of ppGpp in the size of each proteome sector, we consider that Γ induces the transcription of genes of the E sector when too many resources are used by the R sector. In consequence, transcription of the ribosomal sector is suppressed under amino acid limitation [31,57]. In our model, we denote with *η*_*E*_ and *η*_*R*_ the mRNA degradation rates of the E sector and R sector. Basal transcription of genes encoding for proteins belonging to sectors E and R occurs at a maximum rate ${\hat{\theta }}_{E}$ and ${\hat{\theta }}_{R}$, while ppGpp-inducible transcription of sectors E and R is represented by *θ*_*E*_ and *θ*_*R*_, respectively. So, if *ω* and *n* represent Hill parameters describing the interaction between ppGpp and each sector of the proteome, then the temporal dynamics of mRNAs belonging to sectors E and R can be written as

2.2 $$\frac{dm_{E}}{dt}=\theta_{E}\left(\frac{\Gamma^{n}}{\Gamma^{n}+\omega^{n}}\right)+{\hat{\theta }}_{E}\;u(S,X)-\eta_{E}m_{E}$$

and

2.3 $$\frac{dm_{R}}{dt}=\theta_{R}\left(\frac{\omega^{n}}{\Gamma^{n}+\omega^{n}}\right)+{\hat{\theta }}_{R}\;u(S,X)-\eta_{R}m_{R}.$$

Now, let us assume that the synthesis of each metabolite is associated with a cost in terms of energy consumption [41,42]. In particular, we model biosynthesis of the focal amino acid by dividing the E sector into two sub-sectors: E and *y*. Sub-sector E encompasses the majority of catabolic reactions in the cell, and the *y* sub-sector describes the energetic cost of producing amino acid Y and releasing it into the environment (figure 1*a*). Therefore, the translation rate of each mRNA, *κ*_*E*_ and *κ*_*R*_, depends on *J*_*E*_ and *J*_*R*_, which indicate the proportion of ribosomes that are actively translating the mRNAs of each sector. Finally, if *φ* denotes the fraction of sector E that is used for the synthesis of metabolites in sub-sector *y*, and *δ*_*E*_ and *δ*_*R*_ denote degradation rates of proteins from each sector, we can describe the dynamics of different proteome sectors using the following equations:

2.4 $$\frac{dE}{dt}=(1-\varphi )(\kappa_{E}\cdot m_{E})\left(\frac{1-E}{R\cdot J_{E}}\right)-\delta_{E}E,$$

2.5 $$\frac{dy}{dt}=\varphi (\kappa_{E}\cdot m_{E})\left(\frac{1-y}{R\cdot J_{E}}\right)-\delta_{E}y$$

2.6 $$\;\mathrm{and}\;\frac{dR}{dt}=\kappa_{R}\cdot m_{R}\left(\frac{1-R}{R\cdot J_{R}}\right)-\delta_{R}R.$$

By numerically solving equations (2.1)–(2.6), we obtain the temporal dynamics of mRNA and protein concentrations under different environmental conditions. In particular, figure 1*d*,*e* shows a comparison of dynamics exhibited under high- and low-resource conditions (with parameter values described in table 1). Note how, in nutrient-rich conditions, overall production of proteins and mRNAs is promoted, while under resource limitation metabolic activity is reduced and, crucially, ppGpp allocates a larger fraction of the proteome to the E sector. Therefore, under amino acid starvation, growth rate is reduced and synthesis of essential metabolites is enhanced, as illustrated in figure 1*e*.

**Table 1.** Parameters used in the numerical experiments of the multi-scale model for bacterial strains *B*_*x*_ and *B*_*y*_, which are auxotrophic to amino acids *X* and *Y*, respectively.

parameter	value (*B*_*x*_)	value (*B*_*y*_)	description
			*proteome partition model*
*μ*	1 × 10^3^	1 × 10^3^	maximum uptake rate
*k*	50.0	50.0	half-saturation constant
${\hat{\theta }}_{E}$	0.020	0.020	basal maximum transcription rate for the genes on E
${\hat{\theta }}_{R}$	0.08	0.08	basal maximum transcription rate for the genes on R
*θ*_*E*_	20	20	ppGpp inducible transcription rate for the genes on E
*θ*_*R*_	20	20	ppGpp inducible transcription rate for the genes on R
*n*	2.0	2.0	Hill coefficient
*ω*	5.1	5.1	activity threshold for ppGpp in sectors E and R
*κ*_*E*_	60.0	60.0	maximum translation rate for the mRNAs in sector E
*κ*_*R*_	60.0	60.0	maximum translation rate for the mRNAs in sector R
*η*_*E*_	2.4	2.4	degradation rate of the transcripts on E
*η*_*R*_	2.4	2.4	degradation rate of the transcripts on R
*δ*_*E*_	1.7	1.7	degradation rate of molecules from E
*δ*_*R*_	1.7	1.7	degradation rate of transcripts from R
$\delta_{\Gamma }$	0.8	0.8	degradation rate of ppGpp
*J*_*E*_	0.5	0.5	fraction of ribosomes translating proteins from E
*J*_*R*_	0.5	0.5	fraction of ribosomes translating proteins from R
*θ*_*b*_	0.05	0.05	basal ppGpp synthesis rate
*θ*_*i*_	15.2	15.2	induced ppGpp synthesis rate
φ	0.01	0.01	fraction of sector E used for synthesis of amino acid
			*population dynamics model*
*c*	13.95 × 10^9^	13.95 × 10^9^	cell efficiency
*k*_1_	1 × 10^−11^	1 × 10^−11^	growth conversion coefficient
*k*_2_	0.5 × 10^−9^	1 × 10^−9^	amino acid conversion coefficient

## 3. Modelling population dynamics of an auxotrophic consortium

In this section, we will postulate a simple population dynamics model that will allow us to evaluate temporal changes in the abundance of different strains growing in co-culture. First, let us represent with *B*_*x*_(*t*) the density of a bacterial population auxotrophic to amino acid *X* at time *t* ∈ [0, *T*], and with *B*_*y*_(*t*) the density of a strain auxotrophic to amino acid *Y*.

Our model considers that growth rate of strain *B*_*x*_ is a Monod function, $U_{x}(S,X)=\hat{\lambda }\cdot S/\left(K+S\right)$, with growth rate $\hat{\lambda }$ and a half-saturation constant represented with *K*. Previous studies have shown that the concentration of ribosomal proteins is proportional to the cell’s growth rate [38,58], so we use the proteome partition model to obtain $\hat{\lambda }=k_{1}\cdot \lambda$, where *k*_1_ is a proportionality constant and *λ* is computed by interpolating the surface shown in figure 1*c* with the current environmental concentration of glucose (with a concentration denoted with *S*) and amino acid *X* (for which strain *B*_*x*_ is auxotrophic). Then growth rate of strain *B*_*x*_ can be modelled with a growth function *G*_*x*_(*S*, *X*) = *c* *U*_*x*_(*S*, *X*), where *c* denotes a resource conversion parameter into biomass. Similarly, growth rate of strain *B*_*y*_ can be written as *G*_*y*_(*S*, *Y*) = *c* *U*_*y*_(*S*, *Y*).

We also consider that amino acid *X* is produced by strain *B*_*y*_ in surplus and exported into the environment at a rate *β*_*y*_. Similarly, *B*_*x*_ exports into the environment amino acid *Y* at a rate *β*_*x*_. Note that this rate includes both production and export of amino acid into the environment, which we consider to be proportional to the size of sub-sector *y* of E. Therefore, we consider that production of amino acid *Y* by strain *B*_*x*_ occurs at a rate $\hat{\beta_{y}}=k_{2}\cdot \beta_{y}$, where *β*_*y*_ is obtained from the proteome partition model and *k*_2_ is a parameter denoting the conversion of ATP into *Y* molecules (analogous for the production rate of *X*).

**Figure 2.**
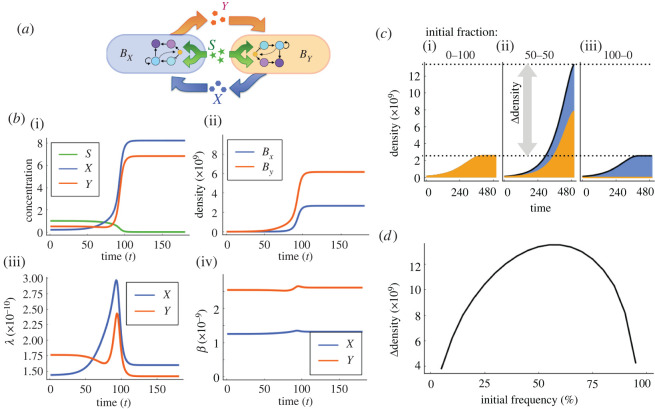
Numerical simulations of population dynamics model. (*a*) Schematic of the population dynamics model for auxotrophic strains (*B*_*x*_ and *B*_*y*_), competing for glucose (*S*, in green) and exchanging essential metabolites (*X* and *Y*, in blue and orange, respectively). Growth rate of each sub-population depends on the availability of resources and can be estimated from the proteome allocation model. (*b*) (i) Numerical results showing the concentration of resources in time: *X* is produced by *B*_*y*_ and consumed by *B*_*x*_, while *Y* is generated by *B*_*x*_ and used as a nutrient by *B*_*y*_. (ii) Bacterial density of each sub-population in an experiment of duration *T* = 180 units of time. (iii) Maximum growth rate (*λ*) obtained from the proteome partition model from the environmental concentrations of resources. (iv) Production and export of amino acids for each strain. (*c*) Growth curves obtained by simulating the model in co-culture (iii), and for each strain grown in isolation ((i) *B*_*x*_ in blue and (ii) *B*_*y*_ in orange). (*d*) To quantify the pair-wise interaction profile, we consider a range of initial population fractions and numerically evaluate the productivity of the co-culture and compare it with respect to that expected in the absence of interaction. Positive interactions are characterized by a concave Δ density curve, indicating that productivity is maximized when both strains are present.

In summary, the population dynamics of the cross-feeding interaction illustrated in figure 2*a* can be described with the following differential equations:

3.1 $$\frac{dS}{dt}=-(U_{x}(S,X)\cdot B_{x}+U_{y}(S,Y)\cdot B_{y})\;S,$$

3.2 $$\frac{dX}{dt}=\hat{\beta_{x}}\cdot G_{y}(S,Y)\cdot B_{y}-U_{x}(S,X)\cdot B_{x},$$

3.3 $$\frac{dY}{dt}=\hat{\beta_{y}}\cdot G_{x}(S,X)\cdot B_{x}-U_{y}(S,Y)\cdot B_{y},$$

3.4 $$\frac{dB_{x}}{dt}=G_{x}(S,X)\cdot B_{x}$$

3.5 $$\;\mathrm{and}\;\frac{dB_{y}}{dt}=G_{y}(S,Y)\cdot B_{y},$$

with initial conditions $\left(S^{0},X^{0},Y^{0},B_{x}^{0},B_{y}^{0}\right)$.

We use a sequential multi-scale modelling approach to integrate both models. First, we compute *λ* and *β* using the proteome partition model for a range of glucose and amino acid concentrations. Then we discretize the interval [0, *T*] in regular sub-intervals [*t*_*i*_, *t*_*i*_ + Δ*t*) and, for each time step, we numerically solve equations (3.1)–(3.5) assuming that growth and production rates are constant during this time interval. From the concentration of each nutrient in the environment at time *t*_*i*_ + Δ*t*, we interpolate the surface shown in figure 1*c* and obtain *λ*_*i*+1_ and *β*_*i*+1_, which are then used to solve the population dynamics model in the next sub-interval. This iterative process allows us to model the dynamics of each sub-population based on instantaneous growth and production rates.

Figure 2*b* shows a numerical realization of the multi-scale model in a low-resource environment (*S*_0_ = 1, *X*_0_ = 0.2, *Y*_0_ = 0.5). As the concentration of amino acids is initially very low, then growth of both populations is limited. However, as the population increases in density, the concentration of *X* and *Y* increases, thus promoting growth of both coexisting sub-populations until *S* is depleted and the population reaches stationary phase. Note how this feedback between the ecological and proteomic models results in values for *λ* and *β* that change in time, as the concentration of resource changes due to consumption and production.

To compare growth in the mixed culture with respect to that observed when each strain is grown in isolation, we consider that the initial population is composed of equal densities of both strains and solve the system forward for *T* units of time. We then compare the final density with that observed in a similar numerical experiment that considers that each strain is growing in isolation. As expected, figure 2*c* shows that mono-cultures present impaired growth while, under the same environmental conditions but with both strains present, the model shows an exponentially growing population that is glucose-limited, instead of limited by amino acids.

We repeated this numerical experiment for a range of initial fractions of both strains and quantified the profile of interaction from the bacterial density observed at the end of the experiment. This allowed us to determine the relative density of the co-culture compared to that expected if there was no metabolic interaction between members of the community. Figure 2*d* shows that the resulting interaction profile is concave, indicating a positive interaction between both strains. In the following section, we will estimate experimentally the profile of interaction for a collection of auxotrophic strains.

## 4. Positive interactions are common in an experimental auxotrophic system

In the past decade, a series of studies have used synthetic communities to study the rules of community assembly under controlled laboratory conditions (see [59–61] for comprehensive reviews on the topic). For instance, a previous study assembled synthetic communities of *E. coli* to understand how communities with different levels of complexity can establish metabolic cross-feeding interactions based on the syntropic exchange of amino acids [29].

Here, we use a similar experimental approach, and use strains of *Escherichia coli* K-12 obtained from the Keio collection [62], with different amino acid auxotrophies conferred by the deletion of the following genes: *glyA*, *hisB*, *ilvA*, *leuB*, *metA*, *pheA*, *thrC*, *trpC* and *tyrA*. Moreover, we use fluorescent markers to measure species abundances at the end of the experiment, thus enabling us to differentiate between mutualism and parasitism in mixed cultures. We validated that each strain exhibits impaired growth unless the amino acid auxotrophy is complemented, either by externally supplementing the amino acid or when paired with a different mutant.

In general, we consider that positive interactions arise when one species promotes the growth of another one (e.g. niche expansion [63,64] or enhancing nutrient availability [15,65]), or negative when an individual in the community inhibits another member’s growth rate (e.g. nutrient sequestration [66,67] or toxin production [68]). Similar to the computational experiments, we characterized the pair-wise interaction profile between strains by measuring the optical density (OD_630_) exhibited by a 50–50 co-culture. By subtracting the mean density of both strains grown in isolation, we obtain the *relative optical density* (ΔOD_630_). Table 2 summarizes the relative optical density obtained for all pair-wise interactions. Note that, although most of them are positive (e.g. Δ*glyA*–Δ*tyrA*, Δ*metA*–Δ*tyrA* and Δ*pheA*–Δ*hisB* have the highest positive relative density), only a few co-cultures exhibit a negative relative density (Δ*pheA*–Δ*thrC* and Δ*glyA*–Δ*hisB*).

**Table 2.** Profile of pair-wise interaction obtained for different pairs of *E. coli* auxotrophic strains.

		mean OD_630_	mean OD_630_			
key	strains	(mono-cultures)	(50–50)	$\Delta {\mathrm{OD}}_{630}$	*α*	*R*^2^
FG	Δ*pheA* − Δ*glyA*	0.4627	0.5417	0.079	0.39273	0.62
FH	Δ*pheA* − Δ*hisB*	0.77335	0.952	0.17865	0.69142	0.759
FI	Δ*pheA* − Δ*ilvA*	0.4064	0.5573	0.1509	0.351	0.666
FL	Δ*pheA* − Δ*leuB*	0.473	0.54	0.067	0.35761	0.749
FM	Δ*pheA* − Δ*metA*	0.466	0.56	0.094	0.39667	0.402
FT	Δ*pheA* − Δ*thrC*	0.49315	0.4858	−0.00735	0.44008	0.100
FW	Δ*pheA* − Δ*trpC*	0.52855	0.6215	0.09295	0.4694	0.750
FY	Δ*pheA* − Δ*tyrA*	0.4615	0.57	0.1085	0.46357	0.585
GH	Δ*glyA* − Δ*hisB*	0.4337	0.4263	−0.0074	0.29576	0.573
GI	Δ*glyA* − Δ*ilvA*	0.44935	0.5477	0.09835	0.36992	0.708
GL	Δ*glyA* − Δ*leuB*	0.4315	0.525	0.0935	0.35719	0.285
GM	Δ*glyA* − Δ*metA*	0.3605	0.471	0.1105	0.39148	0.290
GW	Δ*glyA* − Δ*trpC*	0.3821	0.4733	0.0912	0.35986	0.190
GY	Δ*glyA* − Δ*tyrA*	0.37035	0.6073	0.23695	0.30094	0.796
GT	Δ*glyA* − Δ*thrC*	0.36575	0.475	0.10925	0.29148	0.413
HI	Δ*hisB* − Δ*ilvA*	0.4185	0.477	0.0585	0.4071	0.190
HL	Δ*hisB* − Δ*leuB*	0.4274	0.573	0.1456	0.34599	0.748
HM	Δ*hisB* − Δ*metA*	0.47265	0.5682	0.09555	0.39923	0.681
HT	Δ*hisB* − Δ*thrC*	0.463	0.5415	0.0785	0.39782	0.494
HW	Δ*hisB* − Δ*trpC*	0.44285	0.5277	0.08485	0.39261	0.730
HY	Δ*hisB* − Δ*tyrA*	0.483	0.5495	0.0665	0.41851	0.414
IL	Δ*ilvA* − Δ*leuB*	0.43685	0.5525	0.11565	0.40002	0.886
IM	Δ*ilvA* − Δ*metA*	0.4559	0.5657	0.1098	0.43427	0.778
IT	Δ*ilvA* − Δ*thrC*	0.4489	0.4547	0.0058	0.46806	0.149
IW	Δ*ilvA* − Δ*trpC*	0.4905	0.5755	0.085	0.40843	0.764
IY	Δ*ilvA* − Δ*tyrA*	0.53095	0.587	0.05605	0.46823	0.276
LM	Δ*leuB* − Δ*metA*	0.44635	0.4697	0.02335	0.43427	0.422
LT	Δ*leuB* − Δ*thrC*	0.4596	0.4883	0.0287	0.46806	0.416
LW	Δ*leuB* − Δ*trpC*	0.46885	0.4828	0.01395	0.4099	0.416
LY	Δ*leuB* − Δ*tyrA*	0.57085	0.715	0.14415	0.45674	0.765
MT	Δ*metA* − Δ*thrC*	0.3871	0.4613	0.0742	0.32419	0.379
MW	Δ*metA* − Δ*trpC*	0.4221	0.4565	0.0344	0.31137	0.506
MY	Δ*metA* − Δ*tyrA*	0.365	0.5745	0.2095	0.28458	0.687
TW	Δ*thrC* − Δ*trpC*	0.4894	0.5862	0.0968	0.38874	0.778
TY	Δ*thrC* − Δ*tyrA*	0.45825	0.5655	0.10725	0.34843	0.802
WY	Δ*trpC* − Δ*tyrA*	0.47215	0.5945	0.12235	0.35519	0.035

**Figure 3.**
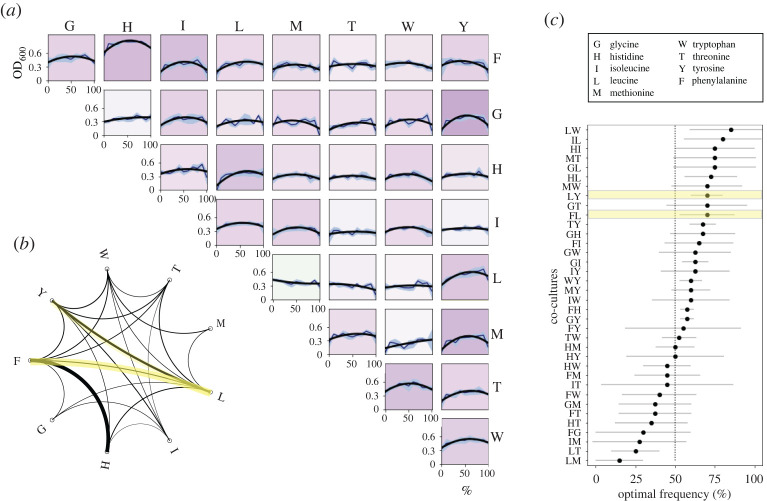
Interaction profiles of *E. coli* strains with different amino acid auxotrophies. Each strain contains a deletion in genes encoding essential metabolites: Δ*glyA*, Δ*hisB*, Δ*ilvA*, Δ*leuB*, Δ*metA*, Δ*pheA*, Δ*thrC*, Δ*trpC* and Δ*tyrA*. (*a*) Each box illustrates growth (measured as OD_630_ after 48 h) in terms of the initial fraction of each strain in the co-culture. Solid line illustrates a quadratic fit (*αx*^2^ + *βx*) and blue areas the standard deviation over the mean. The colour of each box is proportional to the relative density exhibited by a 50–50 co-culture of both strains; white denotes a neutral interaction, while darker shades of purple denote larger values of $\Delta {\mathrm{OD}}_{630}$. In our data, only Δ*leuB*–Δ*metA* exhibits negative relative densities, and is represented in light green. (*b*) Network of pair-wise interactions. Each node represents a mutant strain and the width of the edge is proportional to the strength of the interaction. Only edges with an *α* larger than the mean are shown (highlighted in yellow are auxotroph pairs that will later be analysed in more detail). (*c*) Optimal initial frequency in two-member cross-feeding communities. Each dot represents the initial frequency in co-culture that maximizes density, and error bars denoting the standard deviation over the mean (*N* = 4). Rows correspond to each pair of strains (e.g. if LW has an optimal frequency of 85%, this implies that the co-culture that maximizes OD is composed of 85% of Δ*leuB* and 15% of Δ*thrC*).

Figure 3*a* shows the optical density of all 36 co-cultures with 11 different initial abundances of each strain, from 100% of one strain, to 100% of its partner (see Methods). Although some interactions are positive (e.g. Δ*pheA*–Δ*hisB*, Δ*glyA*–Δ*tyrA*, Δ*leuB*–Δ*tyrA*, Δ*metA*–Δ*tyrA*), not all interaction profiles are concave, suggesting that not all co-cultures can establish a cross-feeding interaction (e.g. Δ*metA*–Δ*glyA*, Δ*thrC*–Δ*ilvA*, Δ*leuB*–Δ*metA*, Δ*leuB*–Δ*trpC*). We performed a quadratic fit (*αx*^2^ + *βx*) to these experimental interaction profiles, and used *α* to quantify the degree of pair-wise interaction, whereby high values of *α* are correlated with enhanced cooperative growth. Table 2 shows the values of *α* obtained for each pair, as well as its corresponding *R*^2^. Figure 3*b* shows an *α*-interaction network, showing that all auxotrophic strains are able to establish a productive consortium with at least another mutant, albeit with variable interaction strengths.

Note that, despite positive interactions being prevalent throughout the network, certain strains were more successful in establishing positive interactions (e.g. Δ*leuB*, Δ*pheA*, Δ*trpC*) than others (e.g. Δ*metA*, Δ*glyA*). This is consistent with previous studies that have shown that the biosynthetic cost of producing an amino acid correlates with the capacity of establishing cross-feeding interactions with other auxotroph strains [29]. In our data, mutant Δ*leuB* has the higher number of cross-feeding interactions, and is also auxotrophic to the most expensive amino acid when considering the cellular abundances of amino acids [69].

**Figure 4.**
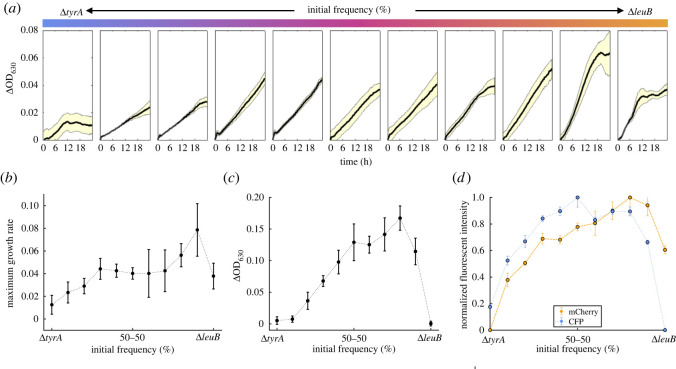
Cross-feeding interaction between Δ*leuB* and Δ*tyrA*. (*a*) Growth curves obtained in LB without antibiotics for different initial fractions of each strain; from a mono-culture of Δ*tyrA* in blue, to Δ*leuB* in orange, with intermediate frequencies represented with a colour gradient. Black line denotes the mean optical density as a function of time in a 24 h experiment, with yellow areas representing the standard error over the mean (*N* = 4 for the co-cultures, *N* = 7 for the mono-cultures). (*b*) Maximum growth rate estimated from 24 h growth curves. Note that growth rate is maximized in co-culture, with strains in isolation exhibiting decreased growth. (*c*) Relative density (measured as ΔOD_630_) as a function of initial abundances of different strains. Error bars represent the mean OD_630_ with standard error (*N* = 8). The shape of this plot indicates a mutualistic profile between Δ*leuB* and Δ*tyrA*, with maximum density achieved around 70% of Δ*leuB*. (*d*) Normalized fluorescence intensity as a function of initial frequency (CFP in blue and mCherry in orange).

To quantify cooperative growth as a function of the relative abundance of each strain, we subtract the final optical density observed for each population with the expected final optical density if there was no metabolic interaction between both strains (the line of additivity, obtained by interpolating the optical density in each mono-culture). Figure 4*a* illustrates the profile of interaction obtained for a co-culture of Δ*leuB* and Δ*tyrA*. A consequence of the biosynthetic costs of producing both amino acids is that the productivity is maximized with Δ*leuB* at 70% and Δ*tyrA* at 30% (relative density at 70–30 is 37% larger than at 50–50). We estimated the optimal initial frequency for all 36 auxotroph pairs and observed that growth of the co-culture was maximized at different initial frequencies, as illustrated in figure 3*c*. It is important to note that the optimal initial ratio is not necessarily the final strain frequency that maximizes productivity of the consortia.

## 5. Cooperative growth is enhanced under amino acid starvation

As we are interested in evaluating how amino acid availability modifies the profile of interaction, we performed high-throughput experiments on a two-dimensional range of externally supplemented amino acids. The basal concentration of each amino acid was obtained from a computational genome-scale metabolic model (iML1515) that provides estimates on the concentration of amino acid necessary to grow a dry weight of bacteria [70,71] (see Methods; 16.91 mg l^−1^ of leucine, 7.15 mg l^−1^ of tyrosine and 8.75 mg l^−1^ of phenylalanine). We performed amino acid checkerboards in triplicate for mono-cultures of Δ*leuB*, Δ*tyrA* and Δ*pheA*, as well as for two co-cultures Δ*leuB*–Δ*tyrA* and Δ*leuB*–Δ*pheA*.

**Figure 5.**
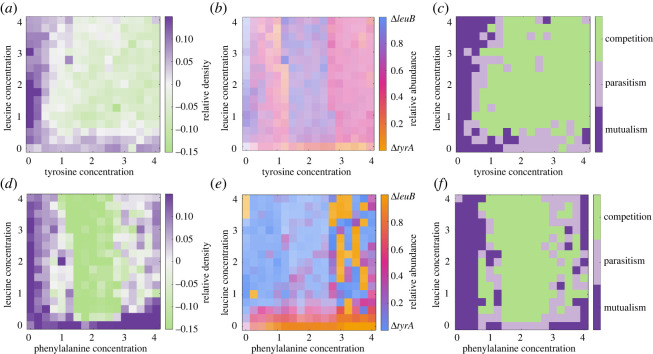
Amino acid checkerboard experiments. (*a*) Matrix of relative densities performed under different amino acid concentrations (leucine and tyrosine, in units of basal concentration). Purple boxes represent high relative density values, white if the interaction is additive, and green boxes correspond to negative relative densities. (*b*) Matrix of the relative abundances estimated using fluorescent intensity, in orange when a majority of the population is Δ*tyrA*, and in blue when the population consists mainly of Δ*leuB* cells. (*c*) Interaction profiles for different amino acid concentrations (mutualism in purple, parasitism in light purple and competition in green). (*d*) Relative density checkerboard obtained by exposing a co-culture of Δ*leuB* and Δ*pheA* to a range of leucine (L) and phenylalanine (F). (*e*) Relative abundance between Δ*leuB* (in blue) and Δ*pheA* (in orange). (*f*) Interaction profile between Δ*leuB* and Δ*pheA* also shows that mutualism (in purple) is established mainly at low amino acid concentrations.

Figure 5*a* illustrates the relative density obtained for Δ*leuB*–Δ*tyrA* growing under different concentrations of both amino acids (from 0 to a fourfold increase in the basal concentration of each amino acid). As anticipated by previous studies, cooperative growth increased when an amino acid was at a low concentration, independently of the concentration of the other metabolite. In particular, our data show that relative densities are maximized at low concentrations of tyrosine and intermediate concentrations of leucine, consistent with leucine being more costly than tyrosine in terms of the number of glucose molecules necessary to produce the amount of amino acids in a cell [69].

To validate that growth is indeed limited by the environmental concentration of the exchanged metabolite, we externally supplemented glucose and amino acid and estimated the resulting relative density. Data are summarized in table 3, showing significant differences between amino acid treatments (*p*-value < 0.001 for Δ*leuB*–Δ*tyrA* co-culture and *p*-value < 0.001 for Δ*leuB*–Δ*pheA* co-culture; Welch two sample *t*-test), but not between glucose treatments (*p*-value > 0.01 for Δ*leuB*–Δ*tyrA* co-culture and *p*-value > 0.001 for Δ*leuB*–Δ*pheA* co-culture; Wilcoxon rank sum test).

**Table 3.** Mean and standard deviation of final OD_630_ of the co-cultures Δ*leuB*–Δ*tyrA* and Δ*leuB*–Δ*pheA*.

co-culture	treatment	mean OD_630_	s.d.
Δ*leuB* − Δ*tyrA*	M9 2 g l^−1^ glucose	0.178250	0.009601
Δ*leuB* − Δ*tyrA*	M9 4 g l^−1^ glucose	0.202250	0.008496
Δ*leuB* − Δ*tyrA*	M9 6 g l^−1^ glucose	0.211000	0.011424
Δ*leuB* − Δ*tyrA*	high amino acids 2 g l^−1^ glucose	0.502000	0.069954
Δ*leuB* − Δ*tyrA*	high amino acids 4 g l^−1^ glucose	0.484750	0.054669
Δ*leuB* − Δ*tyrA*	high amino acids 6 g l^−1^ glucose	0.478750	0.025849
Δ*leuB* − Δ*pheA*	M9 2 g l^−1^ glucose	0.14475	0.05680
Δ*leuB* − Δ*pheA*	M9 4 g l^−1^ glucose	0.207500	0.008732
Δ*leuB* − Δ*pheA*	M9 6 g l^−1^ glucose	0.235250	0.016873
Δ*leuB* − Δ*pheA*	high amino acids 2 g l^−1^ glucose	0.570750	0.52875
Δ*leuB* − Δ*pheA*	high amino acids 4 g l^−1^ glucose	0.480500	0.019033
Δ*leuB* − Δ*pheA*	high amino acids 6 g l^−1^ glucose	0.026818	0.01630

Moreover, to experimentally determine the relative abundances of strains in the consortia, we introduced constitutively expressed genes encoding for different fluorescent markers (mCherry and CFP, as detailed in table 4). This allowed us to estimate the final abundance of each strain from the normalized fluorescent intensity. Indeed, figure 4*d* shows the population exhibits high levels of mCherry fluorescence in co-cultures where Δ*leuB* is dominant, and in cyan for initial frequencies that contain mostly bacterial type Δ*tyrA*.

**Table 4.** List of strains used in this study.

strain ID/Δ gene	amino acid auxotrophy	source
Δ*pheA*	phenylalanine	Keio collection
Δ*glyA*	glycine	Keio collection
Δ*hisB*	histidine	Keio collection
Δ*ilvA*	isoleucine	Keio collection
Δ*leuB*	leucine	Keio collection
Δ*metA*	metionine	Keio collection
Δ*thrC*	threonine	Keio collection
Δ*trpC*	tryptophan	Keio collection
Δ*tyrA*	tyrosine	Keio collection
Δ*leuB* − *mCherry*	leucine	this study
Δ*tyrA* − *eCyan*	tyrosine	this study
Δ*pheA* − *mCherry*	phenylalanine	this study
Δ*leuB* − *eCyan*	leucine	this study

From these normalized fluorescent data, we estimated the relative abundance of each strain in an experiment inoculated with a 50–50 co-culture of both strains and evaluated the interaction between environmental conditions and population structure. For instance, the checkerboard depicted in figure 5*b* suggests that the concentration of tyrosine is an important factor driving the structure of the population; at high concentrations, both strains coexist at similar proportions, while at intermediate tyrosine concentrations, the relative abundance of Δ*leuB* increases with respect to Δ*tyrA*. By contrast, the concentration of leucine appears to be less relevant for the resulting population structure.

Based on the optical density and the relative abundance of each strain, we then estimated the density of each sub-population and compared its growth relative to the corresponding mono-culture under the same environmental conditions. This allowed us to characterize the pair-wise interaction of the community: *mutualistic* when both strains grow better together than in isolation, parasitism when one cell performs better in co-culture, at the expense of the other strain, and *competition* when both mono-cultures grow better than the mixed population. Figure 5*c* shows that competitive interactions are dominant at high levels of both amino acids, while parasitic interactions can be found at intermediate amino acid concentrations. As expected, mutualistic interactions are prevalent at low concentrations of supplemented amino acids, particularly of tyrosine.

Finally, to evaluate the generality of this pattern, we performed another checkerboard experiment with the co-culture Δ*leuB* and Δ*pheA*. Similar to the previous auxotrophic pair, figure 5*d*–*f* shows that, in this case, the region of coexistence appears to be smaller, with Δ*leuB* being more abundant than Δ*pheA* in environments where both amino acids were externally supplemented. Also, as with the Δ*leuB*–Δ*tyrA* pair, mutualistic interactions can be found predominantly in experiments with low amino acid concentrations (in this case, leucine and phenylalanine), while competitive interactions are established at intermediate amino acid concentration (due to glucose becoming the limiting nutrient).

## 6. Stringent response promotes metabolic cooperation, in theory

**Figure 6.**
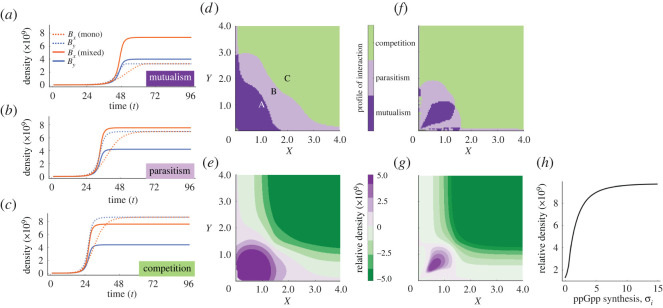
ppGpp modulates the profile of interaction in cross-feeding communities. (*a*) Bacterial densities obtained by simulating the multi-scale model in an environment with low glucose and amino acid concentrations (*S*_0_ = 1, *X*_0_ = 1, *Y*_0_ = 1, with *θ*_*i*_ = 15.2), and inoculated with a 50–50 co-culture (solid lines) or with each strain in isolation (dotted lines). Both strains (*B*_*x*_ and *B*_*y*_, in blue and orange, respectively) exhibit increased growth than expected in isolation (mutualism). (*b*) At intermediate amino acid concentrations (*S*_0_ = 1, *X*_0_ = 1.5, *Y*_0_ = 1.5), only *B*_*y*_ benefits from coexisting with *B*_*x*_ (parasitism). (*c*) In high amino acid environments (*S*_0_ = 1, *X*_0_ = 2, *Y*_0_ = 2), both strains show reduced growth compared to the mono-cultures (competition). (*d*) Profile of interaction determined for a range of both amino acids (mutualism in dark purple, parasitism in light purple and competition in green). (*e*) Relative density for a range of both amino acids (positive interactions and negative interactions in gradients of purple and green, respectively). (*f*) Computational amino acid checkerboard generated with *θ*_*i*_ = 1.5 shows that the region of mutualism and parasitism reduces when limiting induction of ppGpp. (*g*) Relative density under different initial amino acid conditions for strains with reduced ppGpp-induced synthesis. (*h*) Relative density as a function of ppGpp synthesis, *θ*_*i*_, shows that dynamic proteome allocation promotes cooperative growth.

Our experimental data suggest the amino acid availability is the key factor driving the nature of pair-wise interactions. To evaluate if our multi-scale model exhibits qualitatively similar patterns, we simulated the addition of different concentrations of amino acid, and measured the resulting relative density when grown in mono-culture or in a mixed population. Figure 6*a* shows the density of each strain in a low-amino-acid environment. In this case, the benefit of metabolic cross-feeding is high and both strains grow better together than separately. As we increase the concentration of both amino acids, we transition from a parasitic interaction (figure 6*b*) whereby only *B*_*y*_ benefits from growth in co-culture to a competitive interaction where both strains grow better separately than in a mixed population (figure 6*c*).

This transition from mutualism to competition is also clear in the computational checkerboard experiment illustrated in figure 6*d*. As in the experimental data, in environments saturated with amino acids, the benefit of metabolic cross-feeding diminishes and glucose becomes the limiting growth factor. As anticipated by previous studies [2,18,27–29], cooperative growth is maximized at low amino acid concentrations (figure 6*e*). Crucially, it is precisely at low amino acid concentrations where ppGpp reshapes the proteome to suppress growth, and regulate the metabolism to overcome nutrient starvation. In this context, we were interested in evaluating the effect of removing ppGpp-induced transcription at a cellular level in the profile of interaction exhibited by the population.

In our model, this can be achieved by reducing R-induced synthesis of ppGpp (*θ*_*i*_). In particular, we evaluated how the profile of the interaction changed when considering a reduced induction of ppGpp by repeating the computational checkerboard experiment with *θ*_*i*_ = 1.5. As expected, the region of cooperativity presents a significant reduction in area (figure 6*f*). Similarly, the relative density of the co-culture also presents an overall reduction when reducing ppGpp-induction. Figure 6*g* shows that the region of positive values decreases considerably, producing competitive interactions in zones where the interaction was previously mutualistic.

The relevance of ppGpp regulation in the community dynamics is twofold: (1) it reduces growth rate of any fast-growing strain and (2) it allocates more resources for production of the exchanged metabolite. Both of these effects are beneficial for the community, as illustrated by the increase in relative density as a function of *θ*_*i*_ shown in figure 6*h*. In the absence of ppGpp-induced regulation (*θ*_*i*_ = 0), cooperative communities can be driven to collapse due to the over-production of ribosomal proteins and a general increase in gene transcription [30,72]. The community also collapses in environments with low amino acid concentrations if ppGpp regulation overcompensates to nutrient starvation and allocates too many resources for the synthesis of metabolites at the expense of growth. At high amino acid concentrations, the effect of ppGpp in the community dynamics is less evident as, independently of the value of *θ*_*i*_, both strains grow better in isolation than in co-culture.

In principle, it would be possible to test this modelling prediction experimentally, for instance by engineering strains with modified ppGpp synthesis. This could be achieved with either genetic or post-translational modification that modifies the expression of *relA* and *spoT* [32,35]. It is important to mention, however, that previous studies have shown that strains with knockouts in *relA* and *spoT* are auxotrophic to multiple amino acids, mainly due to the role of ppGpp as a regulatory molecule in the transcription of genes involved in amino acid biosynthesis pathways.

## 7. Discussion

A benefit of living in a community is that populations can perform a wide range of functions that are not achievable by any member species alone, as observed in the microbiota of soil [73,74], water [20,75] and the human gut [76]. Metabolic interactions within microbial communities are mediated by the cellular state of each member, but also by the diversity and abundance of each species [77], as well as by the extracellular environmental conditions [78,79]. It has been shown that population dynamics and evolutionary dynamics occur at similar time scales, so drive community dynamics is also susceptible to be tuned by evolution [80,81]. On the one side, cross-feeding interactions can become unstable through the appearance of defectors, although adaptive evolution and precise environmental control present an opportunity for artificially selecting microbial communities with specific functions [61,82–84].

Moreover, recent studies have shown that ecological interactions are significantly modified by the metabolism of individual members of the community [42,85–87]. Bacteria can modify the local environment by secreting toxins or growth-promoting molecules, thus modulating the metabolism of other members of the community. Therefore, metabolic exchange results in a network of pair-wise interactions that can drive the stability and function of polymicrobial communities [29,88], as well as the community dynamics [82,89,90]. Crucially, the competitive or cooperative nature of these interactions depends upon the environmental conditions and the population structure.

For instance, mutualism can be beneficial when a secreted metabolite is toxic for some members of the community but is consumed by other individuals, thus promoting growth of the metabolite-producing population and reducing the toxin’s detrimental effect on the community [9,91]. Other examples of mutualism can be found in spatially explicit [92] and fluctuating environments [93]. In this paper, we focus on studying a ubiquitous form of mutualistic interaction: the exchange of essential amino acids within mixed populations [94–96]. A previous experimental evolution study found that if amino acids are present in the environment in high concentrations, then bacterial populations rapidly lost the ability to autonomously synthesize the supplemented amino acids [97]. This strong selection for the loss of biosynthetic functions is a consequence of auxotrophic mutants exhibiting increased fitness over the prototrophic strain when the focal metabolite is present in the environment [98].

To overcome the complexity of natural microbial communities, previous studies have used synthetic microbial systems to evaluate a community’s capacity to exchange metabolites in controlled environmental conditions [10,46], as well as to evaluate the functional diversity and the role of the environment in community assembly [60,99]. But even simple microbial consortia can present complex ecological dynamics and can be dominated by high-order interactions [59]. Furthermore, reducing community complexity in engineered synthetic consortia can be counter-productive, as a reduction in genetic diversity and functional redundancy can negatively impact the community’s stability. Indeed, in the absence of functional redundancy, removing a member of the consortium can drive the entire population to extinction [21]. We believe this observation is fundamental for designing stable synthetic communities.

In this paper, we combined experiments with multi-scale modelling to evaluate the role of dynamic proteome allocation in the interaction profile exhibited by auxotrophic communities. Both our model and experimental data validate previous studies showing that cooperative interactions are prevalent under nutrient starvation, while competitive interactions are more common in rich environments. Furthermore, numerical simulations of our computational model suggest that suppressing dynamic proteome allocation has the effect of reducing yield of the community, a consequence of the rapid growth and consumption of exchanged metabolites when amino acids are rare. Altogether, we conclude that dynamic proteome allocation is an important factor driving the productivity and interaction profile of cross-feeding microbial communities.

## 8. Methods

### 8.1. Strains and media

All strains used in this study were obtained from the Keio collection [62], each one with different amino acid auxotrophy conferred by the deletion of the following genes: *glyA*, *hisB*, *ilvA*, *leuB*, *metA*, *pheA*, *thrC*, *trpC* and *tyrA*. The genotype of the strains was validated by colony PCR with two different strategies: (1) using for each strain primers located upstream (forward) and downstream (reverse) of the deletion site (band corresponding to the kanamycin resistance cassette) and (2) with the first forward of each gene and the first *k*1 reported in the construction of the collection that it aligns within the kanamycin resistance cassette.

To estimate fluorescent intensities in co-culture, we transformed the strains Δ*tyrA*, Δ*pheA* and Δ*leuB* with a multicopy plasmid assembled by the Golden Gate Cloning technique [100], using the following bioparts from the MoClo kit: pTU1-A-lacZ (vector), pBP-J23100 (promoter), pBP_BBa_B0034 (RBS), pBP-ORF-eCFP (eCyan fluorescent protein) or pBP-ORF-mCherry (mCherry fluorescent protein) and pBP-BBa_B0015 (terminator).

For mono-cultures and co-culture experiments, we selected a single colony of each strain and grew them in LB medium for 16 h with 40 μg ml^−1^ of kanamycin, then harvested by centrifuging at 14 000 r.p.m. for 10 min, washed twice with M9 medium salts (7 g l^−1^ K_2_HPO_4_, 2 g l^−1^ KH_2_PO_4_, 0.58 g l^−1^ Na_3_C_6_H_5_O_7_, 1 g l^−1^ (NH_4_)_2_SO_4_ and 0.1 g l^−1^ MgSO_4_) and suspended in M9 medium with glucose (2 g l^−1^). Before inoculation all strains were adjusted at 0.3 of OD 630 nm (optical density was measured using a BioTek EL×808 plate reader in 96-well plates).

We determined critical amino acid concentrations using the metabolic model iML1515 [70,71]. To determine the amino acid concentration (AC) in milligrams per litre to grow a dry weight of bacteria (RDW) [70,71] we used the following expression: AC [mg l^−1^] = (RDW · (0.295) · ACMW), where ACMW is the amino acid molecular weight.

### 8.2. Pair-wise interaction assays

To evaluate the growth of the strains (mono-cultures) in medium with the amino acid that compensates their auxotrophy we performed kinetic cultures using microplates with 20 μl of cells and 180 μl of M9-glucose media (M9 salts supplemented with 2, 4 or 6 g l^−1^ of glucose and each amino acid: 16.91 mg l^−1^ of leucine, 7.15 mg l^−1^ of tyrosine and 8.75 mg l^−1^ of phenylalanine [70,71]). Each amino acid was supplemented in 0.5, 1, 1.5, 2, 2.5, 3, 3.5 and 4 times the basal concentration for each amino acid (concentrations calculated according to iML1515 model [70]). All plates were incubated at 37°C with continuous shake for 24 h in a BioTek EL×808 plate reader. We measured OD_630_ every 20 min. Using 0 and 3 times the basal concentration of leucine and tyrosine in minimal media with 2, 4 and 6 g l^−1^ of glucose we compared the final OD of our co-culture Δ*leuB*–Δ*tyrA* (50–50) demonstrating growth differences between M9 and M9 supplemented with amino acids (table 3).

To characterize the profile of interaction, we estimated the final density of 36 co-cultures with 11 different initial frequencies between both strains: 200:0, 180:20, 160:40, 140:60, 120:80, 100:100, 80:120, 60:140, 40:160, 20:180 and 0:200 μl. Initial bacterial densities were adjusted in M9-glucose media without amino acids before inoculation, with experiments performed in 96-well plates and incubated at 37°C with 100 r.p.m. orbital shake. Optical density at 630 nm (OD_630_) was measured at different time-points (24, 48 and 72 h). To measure difference in growth rate at the 11 initial frequencies, we evaluated the growth of the co-culture Δ*leuB*–*mCherry* and Δ*tyrA*–*eCyan* in minimal media with 0 amino acids, and measured the optical density every 20 min using a microplate reader (BioTek ELx808). Maximum growth rate was estimated with the slope of a linear fit performed to the exponential phase of the optical density plot (log(OD_630_)).

For checkerboard experiments, we inoculated two 50–50 co-cultures, with Δ*leuB*–*mCherry* and Δ*tyrA*–*eCyan* and Δ*pheA*–*mCherry* and Δ*leuB*–*eCyan*, into a 96-well plate with minimal medium supplemented with a range of leucine and tyrosine and a range of leucine and phenylalanine. For all amino acids, we used concentrations from 0 to 4 times, increasing each 0.25, according to the basal concentration for each amino acid. OD and fluorescence were measured at 0, 24 and 48 h using a fluorescent plate reader (BioTek Synergy H1). To estimate the relative frequency of different strains in co-culture, we used a flow cytometer (Beckman Coulter with 20 000 events in each environmental condition).

### 8.3. Computational experiments

Numerical simulations of the model were performed in Julia using DifferentialEquations.jl [101]. Unless stated otherwise, parameter values used are summarized in table 1. Data analysis was performed in Matlab and Python using standard libraries.

## Data Availability

All data and code used in this study are available in a public repository [102]. Data and relevant code for this research work are stored in GitHub: https://github.com/ccg-esb-lab and have been archived within the Zenodo repository: https://doi.org/10.5281/zenodo.6344915.
